# Artificial Intelligence-Driven Metasurfaces Spanning Multidimensional Light Field Control and Free Space Computing

**DOI:** 10.3390/mi17060667

**Published:** 2026-05-28

**Authors:** Yuchao Wang, Zining Wang, Kaifan Li, Haigang Liang, Xuliang Chai, Zhenhua Wu, Kai Ou

**Affiliations:** 1 Institute of Precision Optical Engineering, School of Physics Science and Engineering, Tongji University, Shanghai 200092, China; 2 MOE Key Laboratory of Advanced Micro-Structured Materials, Shanghai 200092, China; 3 Shanghai Institute of Technical Physics, Chinese Academy of Sciences, Shanghai 200083, China; 4 State Key Laboratory of Transducer Technology, Shanghai Institute of Microsystem and Information Technology, Chinese Academy of Sciences, Shanghai 200050, China; 5 Center of Materials Science and Optoelectronics Engineering, University of Chinese Academy of Sciences, Beijing 100049, China; 6 Shanghai Frontiers Science Center of Digital Optics, Shanghai 200092, China

**Keywords:** meta-optics, artificial intelligence, inverse design, diffractive neural networks, free-space optical computing

## Abstract

Metasurfaces exploit subwavelength scattering elements to manipulate light with a level of flexibility that is difficult to achieve using conventional optical platforms, making them promising building blocks for next-generation photonic systems. Yet the increasing dimensionality of metasurface design spaces and the demand for multifunctional responses have exposed the limitations of traditional intuition-led design approaches. In this Review, we survey the emergence of artificial intelligence (AI)-empowered metasurfaces across three major themes: inverse design, multidimensional optical-field control, and free-space optical computing. We first summarize the fundamental principle of optical field manipulation and the algorithmic approaches to metasurface design, including stochastic optimization, deep neural networks, and generative models, with emphasis on their capabilities in rapid performance prediction and inverse structural discovery. We next discuss artificial intelligence-assisted strategies for engineering multiple spatial, spectral, and polarization degrees of freedom in free space. We then highlight the role of AI-empowered metasurface architectures in optical information processing and computation. Together, these developments point to a powerful framework for integrating machine intelligence with meta-optics, with implications for autonomous photonic systems and high-capacity optical computing.

## 1. Introduction

Metasurfaces are two-dimensional artificial structures composed of subwavelength meta-atoms, which enable unprecedented precise control over the intrinsic dimensions of light waves (phase, amplitude, and polarization) [1,2,3,4,5,6]. Over the past decade, researchers have developed a large variety of compact optical devices with diverse functions, including metalenses [7,8,9], holographic components [10,11,12], virtual/augmented reality systems [13,14,15], etc. Traditional forward design strategies rely on physical intuition and a predefined meta-library, which requires time-consuming parameter scanning. A typical workflow starts with intuition-driven or previously verified structural configurations, followed by solving Maxwell’s equations or analytical formulas to optimize photonic geometries [16,17,18]. However, as metasurfaces evolve toward multidimensional and multifunctional integration, such inefficient design strategies can no longer satisfy the requirements for manipulating full-parameter Jones matrices and complex wavefronts [19].

To address the challenges in metasurface design, AI-empowered inverse design has become a powerful and transformative strategy to overcome the limitations of conventional approaches [20,21]. Inverse design is a goal-oriented methodology that leverages optimization algorithms and aims to solve physical problems using mathematical tools. In the evolution of metasurface inverse design, early efforts were dominated by gradient descent and evolutionary optimization, such as genetic algorithms and particle swarm optimization [22,23]. Typical topology optimization uses the adjoint method to efficiently compute the gradient of an objective function with respect to design variables and gradually optimizes the internal topology to obtain structures that meet the target optical response [24,25,26]. Evolutionary optimization performs global searches over the entire parameter space by simulating natural processes such as evolution and swarm behavior. Nevertheless, both types of methods require massive electromagnetic simulations, incur high computational costs, and struggle to handle high-dimensional and large-scale structural designs [27].

To break through these bottlenecks, the introduction of deep learning has brought revolutionary changes to metasurface inverse design [28,29]. By learning the mapping between structural parameters and optical responses from massive datasets, deep learning enables direct fast-forward prediction and end-to-end inverse design, greatly reducing dependence on full-wave electromagnetic simulations and significantly improving design efficiency [30,31,32,33]. Typical models include multilayer perceptrons and convolutional neural networks, which can accurately fit complex nonlinear responses and rapidly predict key optical parameters, including phase, amplitude, and polarization [34,35,36,37,38]. To further expand the design degree of freedom, generative models, represented by generative adversarial networks, variational autoencoders, and diffusion models, have been developed. These models can directly generate free-form structures and achieve full-dimensional, high-degree-of-freedom light manipulation, which is unattainable using traditional optimization methods [39,40,41,42,43].

The deep integration of metasurfaces and AI has not only improved hardware design efficiency but also advanced free-space optical computing toward high-performance and generalized applications [44,45,46,47,48]. Metasurfaces optimized via AI-empowered inverse design can precisely encode multidimensional optical field information, including phase, amplitude, and polarization, converting complex computational tasks into optical diffraction and modulation processes to achieve low-power, highly parallel linear computing, thereby breaking the speed and power limitations of traditional electronic computing [49,50,51]. Diffractive optical processors based on metasurfaces can perform core computational tasks such as image classification, feature extraction, and Fourier transform in free space. Their parallel processing capability far exceeds that of conventional electronic chips, and they eliminate the need for complex integrated circuits, greatly simplifying optical computing system architectures [52,53,54,55,56].

In recent years, metasurface-based free-space optical computing has entered a new stage of large-scale, high-speed, and multifunctional development [57]. By integrating optical degrees of freedom such as polarization, wavelength, and propagation distance, a single metasurface device can realize independent multitask parallel processing, significantly improving computing capacity and functional integration [58]. This review provides a systematic overview of AI-empowered metasurfaces and their emerging role in free-space optical computing. We first discuss the physical mechanisms of optical-field manipulation with metasurfaces and the inverse-design strategies used to realize high-performance devices, spanning stochastic optimization, deep neural networks, and generative models. We then highlight recent progress in multidimensional optical-field control enabled by AI-assisted methodologies. Finally, we examine the core architectures and applications of metasurface-based free-space optical computing. Together, these advances establish an integrated framework for combining machine intelligence with meta-optics in autonomous photonic systems and high-throughput optical computation.

## 2. Principle of Optical Field Manipulation in Metasurfaces

**Figure 1:**
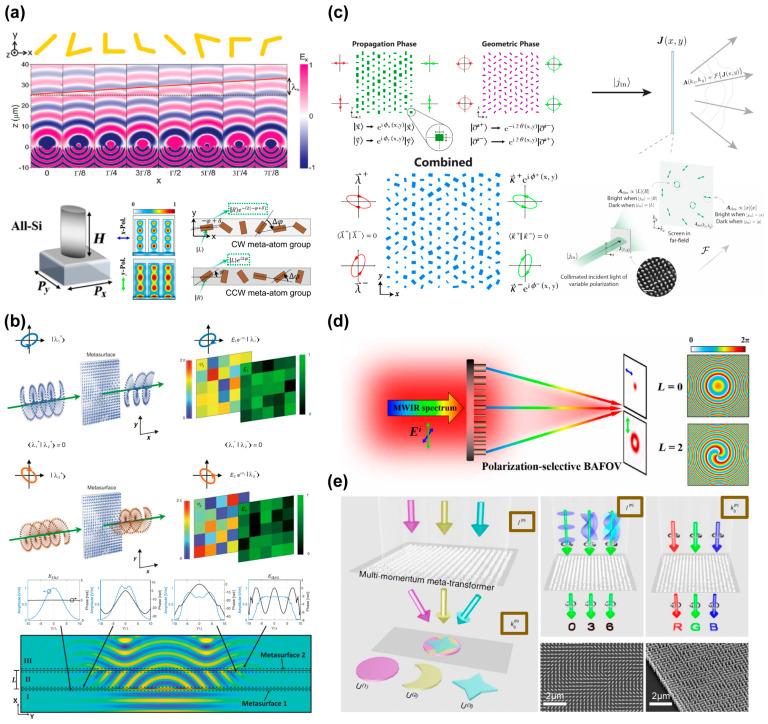
Multidimensional optical field manipulation based on metasurfaces: (**a**) Phase control [59,60,61]. (**b**) Amplitude control [63,64]. (**c**) Polarization control [65,66]. (**d**) Broadband achromatic polarization control [62]. (**e**) Wavelength-multiplexed meta-transformer [67]. Reprinted with permission from Ref. [59]. 2011 AAAS. Reprinted with permission from Ref. [62]. 2020 AAAS. Reprinted with permission from Ref. [63]. 2019 APS. Reprinted with permission from Ref. [65]. 2017 APS. Reprinted from Refs. [60,61,64,67].

Optical metasurfaces are composed of unit cells with subwavelength structures, enabling the manipulation of light’s phase, amplitude, polarization, and dispersion through meticulous engineering of material properties, geometries, and dimensions. Prof. Capasso et al. first proposed the relevant definition of metasurfaces in 2011. As shown in the upper half of Figure 1a, owing to the strong interaction with electromagnetic fields, phase discontinuities were introduced by varying the length and opening angle of V-shaped nano-antennas, which validated the generalized laws of reflection and refraction [59]. Taking the transmissive metasurface shown in the lower-left panel of Figure 1a as an example, we discuss the fundamental principles of phase modulation. The optical response of anisotropic meta-atoms can be expressed as follows [60]:

(1) $$J=R\left(-\theta \right)\left(\begin{array}{cc} A_{mx}e^{i\phi^{mx}} & 0\\ 0 & A_{my}e^{i\phi^{my}} \end{array}\right)R\left(\theta \right)$$

where $R\left(\theta \right)=\left(\begin{array}{cc} cos\theta & sin\theta \\ -sin\theta & cos\theta \end{array}\right)$ denotes the two-dimensional rotation matrix, and $A_{mx}e^{i\phi^{mx}}$ and $A_{my}e^{i\phi^{my}}$ represent the complex transmission coefficients of the meta-atoms under x- and y-polarized illumination, respectively. When the incident light is a circularly polarized (CP) wave ${\hat{e}}_{\pm }=\frac{1}{\sqrt{2}}\left({\hat{e}}_{x}\pm i{\hat{e}}_{y}\right)$, the transmitted field $E_{t}^{\pm }=J{\hat{e}}_{\pm }$ can be derived from Equation (1) as follows:

(2) $$E_{t}^{\pm }=\frac{1}{2}\left(A_{mx}e^{i\phi^{mx}}+A_{my}e^{i\phi^{my}}\right){\hat{e}}_{+}+\frac{1}{2}\left(A_{mx}e^{i\phi^{mx}}-A_{my}e^{i\phi^{my}}\right)e^{\pm i2\theta }{\hat{e}}_{-}$$

Equation (2) reveals two circular polarization components: the co-polarized component sharing the same handedness as the incident CP light without phase delay, and the cross-polarized component with opposite handedness carrying a geometric phase delay of 2θ. By appropriately selecting structural parameters, the condition $A_{mx}\approx A_{my}=A_{0}$ can always be satisfied. Defining $\delta =\phi^{mx}-\phi^{my}$, $\phi^{0}=\phi^{mx}-\phi^{my}$, we obtain the following equation:

(3) $$E_{t}^{\pm }=\frac{A_{0}e^{i\phi_{0}}}{2}\cos\frac{\delta }{2}{\hat{e}}_{\pm }+i\frac{A_{0}e^{i\phi_{0}}}{2}\sin\frac{\delta }{2}e^{\pm i2\phi }{\hat{e}}_{\mp }$$

As shown in Equation (3), the ratio between the two polarization components can be tuned by adjusting the polarization-dependent propagation phases $\phi^{mx}$ and $\phi^{my}$. When δ=±π, the incident light is completely converted into the cross-polarized component (conversion efficiency of 100%), yielding a phase shift of ±2θ. Such a phase shift, dependent on the rotation angle, forms the geometric phase (Pancharatnam–Berry phase). As illustrated in the lower-right panel of Figure 1a, full phase coverage is achieved by rotating the orientation angle θ from 0 to π. In contrast to the geometric phase mechanism, the propagation phase relies on the optical path difference accumulated during electromagnetic wave transmission [61]. The lower-left panel of Figure 1a shows a schematic of the propagation phase meta-atom and the corresponding polarization-dependent magnetic energy density profile, where the propagation phase is realized through the optical path difference generated during wave transmission. The accumulated propagation phase of the dielectric nanopillar is determined by its waveguide mode and is proportional to the nanopillar height [62].

Although flexible phase modulation is crucial for wavefront engineering, achieving high-performance and multifunctional metasurface devices requires the simultaneous control of both phase and amplitude. Most current design strategies typically rely on indirect modulation by introducing losses through polarization conversion, reflection, or coherence. As shown in the upper part of Figure 1b, Liu et al. combined geometric phase and propagation phase and exploited the interference effect between two birefringent TiO_2_ nanopillars to realize independent amplitude and phase encoding for orthogonal circularly or linearly polarized states [64]. Nevertheless, such methods generally suffer from the inherent drawbacks of limited efficiency or energy loss. As depicted in the lower part of Figure 1b, researchers proposed a cascaded metasurface scheme, which employs two phase-only metasurfaces separated by a wavelength-scale distance [63]. The first metasurface reshapes the local power flow distribution via a phase gradient to form the target amplitude profile, while the second metasurface provides phase correction. The proposed strategy allows for fully independent control of amplitude and phase without reflections, absorption, or polarization losses, thus realizing theoretically lossless performance in a bilayer configuration.

As the manipulation of amplitude and phase lays the foundation for complex wavefront engineering, full control over optical fields requires the regulation of polarization. As shown in the left part of Figure 1c, Mueller et al. demonstrated three different phase control strategies using a single-layer metasurface. They integrated the propagation phase and geometric phase in a single-layer metasurface, achieving arbitrary and independent phase control over any pair of orthogonal polarization states for the first time [65]. Building on this achievement, researchers proposed the concept of Jones matrix holography, which generalized this approach, as illustrated in the lower part of Figure 1c. Instead of designing for specific incident polarization states, this work treats the metasurface as a spatially varying Jones matrix transfer function $J\left(x,y\right)$, whose far-field polarization response $A\left(k_{x},k_{y}\right)$ can be derived via Fourier optics [66]. As shown in the figure, the hologram produces a pattern of different polarization-state illustrations, each acting as an analyzer for its depicted state. These advances have elevated metasurface polarization optics from scalar phase modulation to vectorial polarization control.

Dispersion modulation constitutes the fourth dimension for metasurface light-field manipulation, aiming to address the inherent chromatic aberration in diffractive optics or actively exploit wavelength dependence to achieve multifunctional multiplexing. For achromatic design, as presented in Figure 1d, Ou et al. realized polarization-controlled broadband achromatic metadevices in the mid-infrared region using all-silicon birefringent meta-atoms, where chromatic aberration was eliminated through material and structural dispersion engineering of the propagation phase to maintain stable functionality over a broad bandwidth range [62]. On the other hand, as depicted in Figure 1e, Jin et al. demonstrated a strategy for the active utilization of dispersion [67]. The multi-momentum metasurface converter is based on the geometric phase mechanism and integrates orbital angular momentum with linear momentum. Under illumination with right-circularly polarized vortex beams, the OAM meta-transformer generates distinct images at the same plane with left-circular polarization. When illuminated at different wavelengths, the single metasurface can reconstruct the letters “R”, “G”, and “B” as well as color holographic images, thereby significantly increasing the information capacity.

## 3. Inverse Design Methods for Metasurfaces

### 3.1. Gradient-Based and Evolutionary Optimization

**Figure 2:**
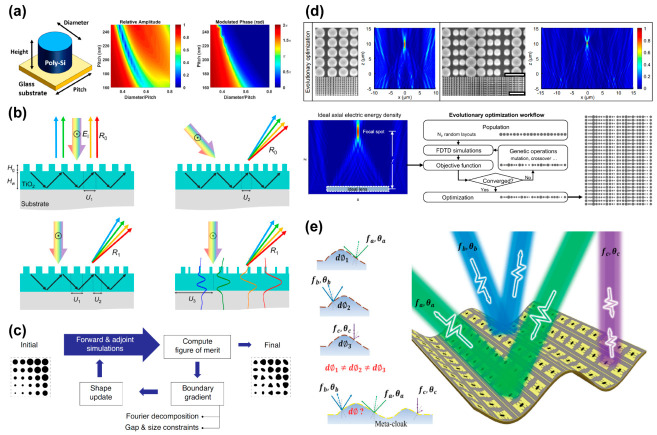
Numerical optimization methodologies for metasurfaces: (**a**) Library-based method [75]. (**b**) Topology optimization [76]. (**c**) Shape optimization [68]. (**d**) Genetic algorithm [77]. (**e**) Evolutionary games-assisted synchronization metasurface [78]. Reprinted with permission from Ref. [75]. 2018 American Chemical Society. Reprinted with permission from Ref. [78]. 2024 John Wiley and Sons. Reprinted from Refs. [68,77].

As a classic strategy for metasurface inverse design, numerical optimization reformulates the design task into a mathematical optimization problem, in which structural parameters are iteratively optimized to drive the device’s optical response toward the target. Initially, metasurface design was primarily based on libraries of meta-atoms. Specific geometric parameters (e.g., nanopillar diameter) were pre-calculated to derive their functional relationships with phase and amplitude responses. The metasurface was then divided into independent unit cells for corresponding mapping, as shown in Figure 2a [68]. However, this method neglects the near-field coupling between unit cells, which limits the performance of complex wavefront manipulation. To address this issue, gradient-based optimization methods, especially topology optimization, realize the continuous discretization of the design space and efficiently calculate the gradients of the objective function with respect to thousands of design variables using the adjoint algorithm [69]. By utilizing the adjoint algorithm to efficiently compute gradients for thousands of design variables, a randomly initialized refractive index distribution can converge to a final binarized geometry [70,71,72,73,74]. For example, Dainese et al. applied this method to design a resonant waveguide grating on a titanium dioxide substrate. The device achieved ultra-narrowband color selectivity with a quality factor as high as Q = 1362, as illustrated in Figure 2b [68].

Unlike topology optimization, the shape optimization proposed by Dainese et al. focuses on the smooth evolution of existing geometric features. It achieves high efficiency while ensuring manufacturing robustness via Fourier decomposition of the surface gradient, as shown in Figure 2c [68]. When the design objective involves a complex space with non-differentiable functions or design spaces prone to local optima, evolutionary optimization (e.g., genetic algorithms, particle swarm optimization) exhibits unique advantages through global search inspired by natural evolutionary processes. In Figure 2d, Cai et al. have inversely designed an ultra-thin non-local metasurface based on genetic algorithms, successfully designing an ultra-thin metalens with a thickness of only 115 nm, which achieved high-efficiency focusing in the visible light region [77]. In addition, to tackle the coupling challenges in dynamic multisource environments, Huang et al. proposed synchronization metasurfaces optimized via evolutionary game algorithms, decoupling the correlation between incident directions and frequencies, enabling simultaneous invisibility cloaking, as shown in Figure 2e [78]. In summary, the aforementioned numerical optimization strategies provide a mathematically rigorous framework for achieving multifunctional integration, facilitating the discovery of non-intuitive nanostructures. These strategies necessitate costly full-wave electromagnetic simulation iterations from scratch for each new target. With the growing demand for larger-scale and more functional meta-devices, such prohibitive time costs and model non-reusability have become bottlenecks restricting their application in metasurface design.

### 3.2. Deep Neural Networks

**Figure 3:**
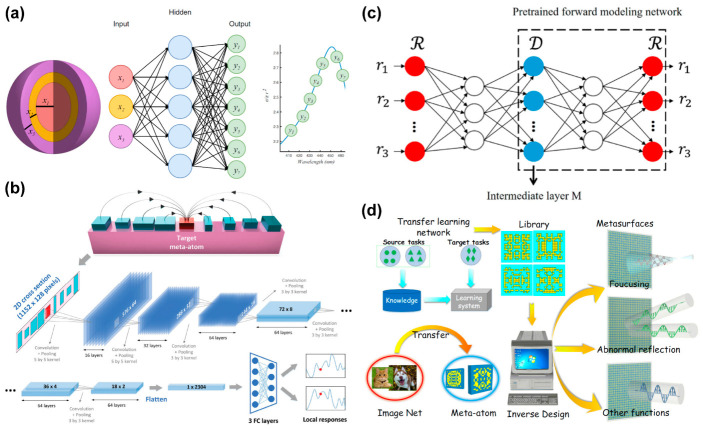
Deep learning-empowered metasurface modeling and design: (**a**) Neural network architecture for approximating light scattering from multilayer nanoparticles [81]. (**b**) Deep convolutional neural network for predicting non-local near-field coupling effects in large-area metasurface arrays [82]. (**c**) Tandem neural network architecture incorporating physical constraints to overcome the non-uniqueness issue in inverse design [83]. (**d**) Phase-to-pattern inverse design paradigm based on transfer learning [84]. Reprinted with permission from Ref. [81]. 2018 AAAS. Reprinted with permission from Ref. [82]. 2021 John Wiley and Sons. Reprinted with permission from Ref. [83]. 2018 American Chemical Society. Reprinted from Ref. [84].

Although the aforementioned numerical optimization algorithms perform excellently in unit cell optimization, each search for a new target requires iterative calculations from scratch and is extremely dependent on the high computational cost of full-wave simulations. As device size and complexity increase, these methods become exceptionally slow. In contrast, deep neural networks construct data-driven surrogate models that can perform efficient real-time inference after training, greatly improving the efficiency of inverse design [26,31,79,80]. In Figure 3a, Peurifoy et al. proposed a method using artificial neural networks to approximate light scattering by multilayer nanoparticles, achieving orders of magnitude faster simulations than conventional methods once trained [81]. This approach enables high-precision approximation with only a small sample of data. To address the issue of neglecting near-field coupling in aperiodic arrays in traditional designs, An et al. further introduced deep convolutional neural networks. By taking the physical specifications of the target unit and its neighboring structures as joint inputs, they successfully quantified nonlocal interactions, enabling large-area metasurfaces to perform precise phase compensation within milliseconds and significantly enhancing the diffraction efficiency of devices, as shown in Figure 3b [82].

However, training deep neural networks for inverse design faces fundamental challenges arising from the pervasive non-uniqueness issue in inverse scattering problems, which often leads to poor convergence of the network [85]. To tackle this, Liu et al. proposed a tandem neural network architecture, as shown in Figure 3c [83]. This framework effectively overcomes data inconsistency by coupling a pre-trained forward physical network to provide physical constraints for the inverse design network, ensuring stability during the inverse matching of complex nanostructures. Furthermore, since deep learning relies on massive training datasets, transfer learning has increasingly been employed in model training in recent years. Transfer learning allows a pre-trained model to share its knowledge and experience with a target network, thereby improving performance in different scenarios and alleviating data scarcity [86,87]. As shown in Figure 3d, Zhu et al. proposed a “phase-to-pattern” design paradigm based on transfer learning. This network enables the monolithic, integrated generation of $2^{8\times 8}$ meta-atom array structures directly from input phase profiles with an accuracy of up to 90% [84]. These advances demonstrate that deep learning not only accelerates the development of individual devices but also promotes the advancement of metasurface design toward intelligence and large-scale implementation.

### 3.3. Generative Networks

**Figure 4:**
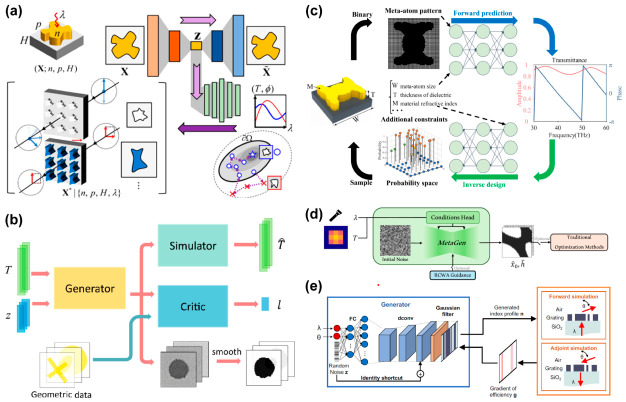
Generative models for metasurface inverse design: (**a**) End-to-end deep generative framework incorporating fabrication constraints and integrating autoencoders with fully connected networks [39]. (**b**) Customized spectrum-driven metasurface pattern generation model based on GANs [91]. (**c**) Tandem Generative Network incorporating physical constraints from a forward prediction network [92]. (**d**) High-fidelity inverse design of far-field spatial power distributions using Denoising diffusion probabilistic models [93]. (**e**) Physics-driven GLOnet integrating electromagnetic simulators into the loss function [94]. Reprinted with permission from Ref. [39]. 2023 American Chemical Society. Reprinted with permission from Ref. [91]. 2018 American Chemical Society. Reprinted with permission from Ref. [92]. 2025 American Chemical Society. Reprinted with permission from Ref. [94]. 2019 American Chemical Society. Reprinted from Ref. [93].

While discriminative neural networks perform excellently in response prediction, they still face challenges such as insufficient generalizability and training non-convergence when dealing with the “one-to-many” mapping in inverse design and generating free-form structures that transcend predefined parameter libraries. To overcome these limitations, generative models offer a new paradigm for the development of high-performance meta-devices by learning the probability distribution of the design space [26,27,88,89,90]. As shown in Figure 4a, Tanriover et al. proposed an end-to-end deep generative framework. This shape generation method, which considers fabrication constraints, constructs diverse datasets and designs a forward model integrating autoencoders with fully connected networks [39]. As design requirements evolve toward customized spectra, generative adversarial networks (GANs) significantly improve the mapping quality from target functions to physical patterns through the zero-sum game between the generator and the discriminator. As shown in Figure 4b, this model can systematically generate candidate patterns with extremely high matching degrees according to any user-defined target spectrum, greatly shortening the conversion cycle from functional requirements to physical structures [91].

However, GANs often suffer from a lack of physical robustness when handling meta-atoms with extremely high degrees of freedom, leading to generated results whose optical responses deviate from expectations. To this end, researchers introduced the tandem generative network, which solves the non-uniqueness problem in inverse design and ensures that the generated complex meta-atoms not only have diverse morphologies but also meet strict optical response standards, as illustrated in Figure 4c [92]. Recently, denoising diffusion probabilistic models have begun to replace traditional GANs in diffractive metasurface design due to their superior generation quality and stability [41]. Hen et al. utilized a conditional diffusion model to achieve precise inverse control over complex far-field spatial power distributions, as illustrated in Figure 4d [93]. By learning the complex nonlinear relationships between geometric shapes, structural heights, and far-field scattering patterns, this method can predict the optimal meta-unit configuration according to specific target far-field distributions, demonstrating stronger robustness and generation detail restoration than GANs. In summary, variational autoencoders effectively learn latent distributions but often lack structural precision. GANs offer fast generation speeds yet suffer from instability and non-convergence when handling high-degree-of-freedom structures. Diffusion models, despite higher computational cost, excel in structural diversity, fidelity, and precise far-field spatial control. Recently, physics-informed neural networks (PINNs) have emerged as a powerful paradigm that embeds governing physical laws, such as Maxwell’s equations, directly into the loss function during training. This approach ensures physically consistent predictions and improves generalization for metasurface inverse design. To further integrate physical mechanisms directly into the optimization process, Jiang et al. proposed a physics-driven neural network global optimization strategy (GLOnet), as shown in Figure 4e [94]. GLOnet uses analytical gradients calculated via the adjoint method to backpropagate through the network, thus enabling training without requiring pre-computed datasets or discriminative networks. By taking target deflection angles and wavelengths as conditional inputs, the model can efficiently generate optimized structural configurations for specific parameters over a broad parameter range [95]. In summary, driven by diverse optimization algorithms, metasurface inverse design has evolved into a highly automated framework powered by AI. These methods not only exhibit outstanding performance in solving high-dimensional nonlinear spatial mapping but also significantly reduce the computational cost of electromagnetic simulations through the collaboration of discriminative and generative models. Furthermore, these optimization algorithms enable metasurfaces to demonstrate great potential in full-parameter manipulation and multifunctional integration, laying a solid algorithmic foundation for complex vectorial optical field regulation and high-performance free-space optical computing systems.

## 4. AI-Empowered Metasurfaces for Optical Field Control and Optical Information Processing

### 4.1. AI-Empowered Metasurfaces for Free-Space Optical Field Control

**Figure 5:**
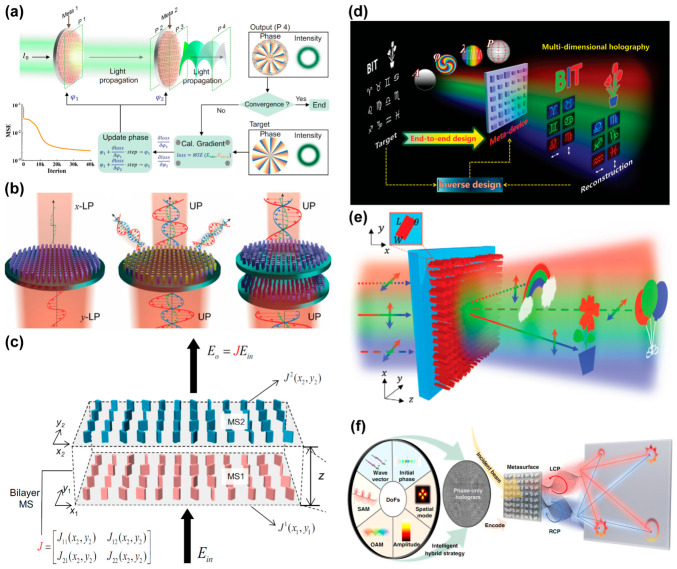
Multidimensional free-space optical field modulation: (**a**) Cascaded phase-only metasurface architecture for generating high-purity vortex beams with high topological charges [99]. (**b**) Cascaded modulation scheme utilizing bilayer birefringent meta-optical arrays for independent complex-amplitude control [100]. (**c**) Bilayer metasurface system to achieve complete control over all eight independent degrees of freedom of the full-parameter Jones matrix [49]. (**d**) End-to-end inverse design for 12-channel multidimensional multiplexed holography [101]. (**e**) Neural network-assisted end-to-end framework leveraging dispersive full-parameter Jones matrices for multi-wavelength, multi-depth holography under non-orthogonal polarizations [102]. (**f**) Intelligent hybrid modulation strategy for simultaneous and on-demand synergistic control of six physical dimensions on a single-layer device [103]. Reprinted with permission from Ref. [100]. 2022 American Chemical Society. Reprinted with permission from Ref. [101]. 2024 John Wiley and Sons. Reprinted with permission from Ref. [102]. 2025 John Wiley and Sons. Reprinted from Refs. [49,99,103].

In the field of free-space optical field manipulation, metasurfaces have demonstrated unprecedented capabilities for integration and multidimensional control, transcending the limitations of conventional optical components. As application scenarios evolve from basic wavefront shaping to complex full-space vector control, researchers have shifted their focus from the response design of individual meta-atoms to system-level collaborative optimization. The introduction of inverse design and neural networks has provided effective solutions for addressing the complex nonlinear coupling among multidimensional physical quantities [96,97,98]. In the research pursuit of optical field manipulation with extreme mode purity, eliminating the intrinsic coupling between phase and amplitude among meta-atoms is a key bottleneck. Cascaded phase metasurfaces provide an effective path for independent manipulation of intensity distribution and phase matching through spatial wavefront transformation. Mei et al. proposed a cascaded architecture optimized based on optical neural networks, using the first metasurface for intensity reconstruction and the second for phase compensation, as shown in Figure 5a [99]. This step-by-step modulation approach overcomes the difficulty that single-layer devices struggle to simultaneously achieve complex amplitude modulation, successfully realizing the generation of high-purity Laguerre–Gaussian modes. When the control requirements extend to all basic properties of light (amplitude, phase, and polarization), traditional single-layer transmission matrices, due to their inherent symmetry limitations, make it difficult to achieve truly independent complex-valued optical field manipulation. To break this physical constraint, a cascaded bilayer birefringent meta-optical system was introduced, utilizing spatial heterogeneity to further decouple orthogonal polarization states. As shown in Figure 5b, Zheng et al. leveraged a bilayer architecture and end-to-end inverse design to achieve high-efficiency vectorial holography and mode multiplexing through wavefront redistribution between the two layers [100]. Single-layer metasurfaces are constrained by planar symmetry, allowing at most the modulation of three components of the Jones matrix. To overcome this restriction and achieve simultaneous control over all four components, Bao et al. experimentally observed a full-parameter Jones matrix with eight independent degrees of freedom for the first time in a bilayer cascaded system [49]. By performing global optimization of the spatially varying Jones matrix via a gradient descent algorithm, they achieved complete control over all informational degrees of freedom within the Jones matrix, as illustrated in Figure 5c.

Although multi-layer cascading provides extremely high degrees of freedom, simplifying the design process and reducing reliance on complex meta-atom libraries remain urgent challenges to be addressed in practical applications. The end-to-end inverse design method avoids complex physical derivations and directly performs a global search for meta-atoms based on target functionalities. As shown in Figure 5d, Yin et al. proposed an end-to-end design framework, realizing 12-channel multidimensional multiplexed holography, including wavelength, plane, and polarization, using meta-atoms with only two degrees of freedom [101]. Addressing more complex issues such as broadband dispersion, multi-depth, and non-orthogonal polarization control, Chi et al. further achieved multi-wavelength and multi-depth holographic display under non-orthogonal polarization using a dispersive full-parameter Jones matrix through a neural network-assisted end-to-end framework, as depicted in Figure 5e [102]. With the deepening of research, achieving full-dimensional collaborative manipulation on a single compact device is currently the goal of optical field manipulation. As shown in Figure 5f, Zhou et al. proposed an intelligent hybrid control strategy [103]. This work successfully manipulated six dimensions of degrees of freedom simultaneously on a single-layer metasurface: wave vector, initial phase, spatial mode, amplitude, orbital angular momentum, and spin angular momentum. This 288-dimensional high-dimensional control space constructed on a single-layer device demonstrates that metasurfaces have evolved into all-purpose platforms capable of processing complex, multidimensional photonic information, offering unlimited possibilities for free-space optical computing and advanced display technologies.

### 4.2. Metasurface-Enabled Free-Space Optical Computing Architectures

**Figure 6:**
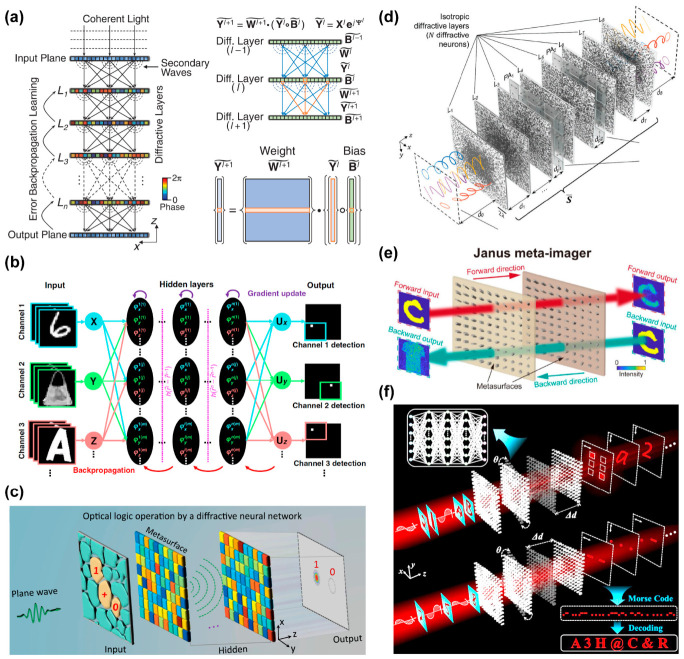
Metasurface-based free-space optical computing: (**a**) Architecture of D^2^NN for all-optical object classification via passive layers [47]. (**b**) Polarization-multiplexed diffractive networks for parallel recognition of heterogeneous tasks [109]. (**c**) Spatially encoded diffractive logic gates performing all-functional optical Boolean operations [48]. (**d**) universal polarization transformer enabling spatial programming of arbitrary polarization scattering matrices [55]. (**e**) Janus meta-imager exploiting asymmetric frequency responses for direction-dependent image transmission [110]. (**f**) Multi-DoF integrated processor synergistically scheduling polarization, position, and rotation for high-capacity, secure computing [58]. Reprinted with permission from Ref. [47]. 2018 AAAS. Reprinted with permission from Ref. [48]. 2023 John Wiley and Sons. Reprinted with permission from Ref. [58]. 2025 John Wiley and Sons. Reprinted from Refs. [55,109,110].

Optical neural networks leverage light–matter interactions to perform analog computing. In diffractive deep neural networks (D^2^NN), spatially structured passive diffractive layers modulate the incident optical field; the propagation between layers mimics weighted connections, while the intensity at each diffractive node acts as a nonlinear-like activation. Thus, an input optical image is transformed layer by layer into an output optical field that directly accomplishes tasks such as image classification or matrix operations without electronic processing. Metasurfaces, with their subwavelength manipulation capabilities, provide a physical foundation for directly implementing such complex mathematical operations in free space [104]. By cascading metasurfaces as spatial light modulators or diffractive layers, the propagation of an optical field can be regarded as performing a series of linear or nonlinear physical transformations. This computing mode at the speed of light not only features ultrahigh parallel processing capacity but also holds the potential for fast, large-scale parallel computing with low power consumption [105,106,107,108]. Lin et al. pioneered the physical architecture of D^2^NN, as illustrated in Figure 6a [47]. D^2^NN employs multilayer passive diffractive layers as physical operators, with each pixel acting as a neuron, enabling feature extraction and classification of complex images at the speed of light. To further enhance the information processing capacity of a single device, Luo et al. introduced the polarization multiplexing dimension, as shown in Figure 6b [109]. Through the decoupled control of orthogonal polarization states in sub-wavelength meta-atoms, a single network can execute digit recognition and fashion product classification tasks in parallel, extending both the capability and computational efficiency of metasurface computing.

While classification tasks demonstrate remarkable inference capabilities, the core of advancing optical computing toward universality lies in executing fundamental mathematical and logic operations. Qian et al. demonstrated all-functional optical logic operators enabled by spatial encoding, as shown in Figure 6c, which maps Boolean inputs to specific wavefront states to achieve high-contrast logic gates without precise interference control, providing the underlying logic for universal photonic processors [55]. Li et al. proposed a full-space polarization transformer, as presented in Figure 6d [48]. Polarizer arrays are embedded between isotropic diffractive layers, and tens of thousands of diffractive features are optimized via deep learning to achieve universal polarization transformation. With in-depth research on diffractive neural networks, researchers have begun to use neural networks to learn complex physical properties to realize special transmission functions. Chong et al. developed a Janus meta-imager, as shown in Figure 6e, which learns the asymmetric spatial frequency distributions to enable direction-dependent image reconstruction, offering new mechanisms for unidirectional perception and physical-layer security [110]. Ultimately, to address the demands for high task capacity and multifunctional integration, Zhang et al. constructed a multi-degree-of-freedom integrated processor, as illustrated in Figure 6f [58]. By synergistically scheduling polarization, spatial position, rotation, and distance, the system integrates classification, logic operations, and Morse-coded encryption into a single platform. These advancements signify that metasurface-based diffractive computing is evolving from fundamental validation toward intelligent photonic systems with high capacity, enhanced security, and universal mathematical processing capabilities.

## 5. Conclusions

As a revolutionary planar photonic platform, metasurfaces have thoroughly reshaped the technological paradigms of optical field manipulation and free-space optical computing by virtue of subwavelength structural engineering and multidimensional electromagnetic wave regulation. Over the past decade, AI has become an indispensable core tool for the development of meta-optics. This review first introduces the mechanisms of optical field modulation with metasurfaces [21,29,111,112,113] and then, starting from the algorithms, compares the advantages of numerical optimization, discriminative networks, and generative models in solving different challenges. Next, it elaborates on how AI-empowered metasurfaces unlock the ultimate manipulation of all photonic degrees of freedom (phase, amplitude, polarization, spatial position, etc.) in free space, demonstrating the capability of metasurfaces for multidimensional optical field control. Finally, we highlight the cutting-edge breakthroughs of D^2^NN in free-space optical computing, covering system integration ranging from basic image classification and multi-task multiplexing to universal logic operations and multidimensional encrypted transmission. Collectively, these studies reveal that metasurfaces have evolved beyond mere static modulation devices into intelligent physical platforms capable of mathematical operations.

Despite remarkable progress, the large-scale commercialization of AI-empowered metasurfaces still faces severe challenges. First are design and manufacturing constraints: meta-atoms typically feature high aspect ratios and tiny feature sizes, where minimal processing tolerances can lead to severe performance degradation, and current CMOS-compatible fabrication processes still struggle to achieve real-time independent control of megapixel-scale elements. Second, in terms of algorithms and data, deep learning models suffer from the “curse of dimensionality” when dealing with ultra-high-degree-of-freedom aperiodic structures, and their reliance on massive simulation datasets results in extremely high computational costs. Furthermore, at the system level, all-optical computing systems still lack efficient, low-power nonlinear activation functions, and subwavelength alignment accuracy between cascaded layers represents a critical bottleneck limiting computational precision.

With the development of free-space optical computing and breakthroughs in fabrication technology, this field is entering a new stage characterized by precision, dynamicization, and interdisciplinary integration. Li et al. implemented model-free optimization directly on hardware using proximal policy optimization reinforcement learning, effectively overcoming the challenges of experimental noise and simulation-to-reality mismatch [114]. Tzarouchis et al. developed a reconfigurable wave-based analog computing machine capable of physically executing non-stationary iterative algorithms, such as Newton’s method and the Lagrange multiplier method, transcending the previous limits of simple linear optical operations [115]. To address the stringent alignment challenges in multilayer systems, Wang et al. proposed a misalignment-resilient PF-D^2^NN architecture by introducing phase-filtering operators into the modulation layers, enabling high-fidelity image generation even with alignment errors exceeding 5 pixels [116]. In summary, the convergence of metasurfaces and AI is giving rise to a new research paradigm. As manufacturing processes advance toward high precision and large areas, and the autonomous decision-making capability of AI agents in complex design tasks continues to improve, we are confident that AI-empowered metasurfaces will become a key pillar of all-optical visual perception, 6G mobile communication, and edge computing systems.

## Data Availability

No new data were created or analyzed in this study.
